# Betatrophin Levels Are Related to the Early Histological Findings in Nonalcoholic Fatty Liver Disease

**DOI:** 10.3390/metabo11070425

**Published:** 2021-06-28

**Authors:** Alper Sonmez, Teoman Dogru, Cemal Nuri Ercin, Halil Genc, Gurkan Celebi, Hasan Gurel, Serkan Tapan, Ali Fuat Cicek, Cem Barcin, Cem Haymana, Ali Kirik, Manfredi Rizzo

**Affiliations:** 1 Department of Endocrinology and Metabolism, Gulhane School of Medicine, University of Health Sciences, Ankara 06010, Turkey; 2 Department of Gastroenterology, School of Medicine, Balikesir University, Cagis, Balikesir 10145, Turkey; teomandogru@yahoo.com; 3 Department of Gastroenterology, Gulhane School of Medicine, University of Health Sciences, Ankara 06010, Turkey; cnercin@hotmail.com (C.N.E.); dr_gurkancelebi@yahoo.com (G.C.); 4 Department of Gastroenterology, Batigoz Hospital, Balcova, Izmir 35330, Turkey; zhgenc@yahoo.com; 5 Department of Gastroenterology, University of Health Sciences, Samsun Training and Research Hospital, Samsun 55090, Turkey; hgurel55@yahoo.com; 6 Section of Biochemistry, ENA Laboratory, Ankara 06680, Turkey; serkantapan7@yahoo.com; 7 Department of Pathology, Gulhane School of Medicine, University of Health Sciences, Etlik, Ankara 06010, Turkey; afcicek@gmail.com; 8 Department of Cardiology, Gulhane School of Medicine, University of Health Sciences, Ankara 06010, Turkey; cem.barcin@sbu.edu.tr; 9 Department of Endocrinology and Metabolism, Gulhane Training and Research Hospital, University of Health Sciences, Ankara 06010, Turkey; cemhaymana@hotmail.com; 10 Department of Internal Medicine, School of Medicine, Balikesir University, Cagis, Balikesir 10145, Turkey; alikirik87@hotmail.com; 11 Division of Endocrinology, Diabetes and Metabolism, Department of Medicine, University of South Carolina, Columbia, SC 29208, USA; manfredi.rizzo@unipa.it; 12 Department of Health Promotion, Mother and Child Care, Internal Medicine and Medical Specialties (PROMISE), University of Palermo, 90133 Palermo, Italy

**Keywords:** betatrophin, nonalcoholic fatty liver disease, glucose intolerance, insulin resistance

## Abstract

Betatrophin, a liver hormone, regulates glucose and lipid metabolism. We investigated the betatrophin levels in nonalcoholic fatty liver disease (NAFLD) and searched for any relationship with histological severity and metabolic parameters. Fifty males with NAFLD [Nonalcoholic Steatohepatitis (NASH) (n = 32); non-NASH (n = 18)] and 30 healthy controls were included. Plasma betatrophin was measured by ELISA method. Insulin sensitivity was assessed by HOMA-IR index. Histological features were scored by the semi quantitative classification and combined as the NAFLD activity score (NAS). Betatrophin levels in the non-NASH group were significantly higher than the controls. Betatrophin was positively correlated to the age, waist circumference, total cholesterol, triglycerides, LDL cholesterol, glucose, insulin, HOMA-IR index and gamma glutamyl transpeptidase levels, and negatively correlated to the steatosis and NAS. In the stepwise linear regression analysis, the triglyceride (β = 0.457, *p* < 0.001), glucose (β = 0.281, *p* = 0.02) and NAS (β = −0.260, *p* = 0.03) were the independent determinants of betatrophin. Betatrophin levels are higher in the early stages of NAFLD and tend to decrease when the disease progresses. This could be an important preliminary mechanistic finding to explain the increased frequency of glucose intolerance during the course of NAFLD.

## 1. Introduction

The liver hormone betatrophin (ANGPTL8, Lipasin), which regulates glucose and lipid metabolism, has been identified by different groups, in different names [1,2,3,4]. The preliminary report mentioning that betatrophin induces beta cell proliferation [1] was not replicated in the later studies [4,5]. However, it is still conceivable that Betatrophin may play a role in other ß-cell functions [6]. Therefore, searching for the role of betatrophin in the pathogenesis of chronic non-communicable diseases is very exciting as it may herald the emergence of promising new treatment modalities.

So far, the mechanism of the effect of betatrophin in physiological and pathological conditions is not clearly known. Also, there is very few and controversial data about the role of betatrophin in chronic metabolic disorders such as obesity, metabolic syndrome and diabetes mellitus (DM). Some studies reported high betatrophin levels in both type 1 [7] and type 2 [8,9,10,11] DM. However, unaltered [12] or low levels in diabetics were also described [13]. There is also controversial data in terms of the relationship between the serum lipids, visceral adiposity and insulin resistance [7,8,9,10,11,12,13].

Nonalcoholic fatty liver disease (NAFLD) is the most common liver disorder in the modern world [14]. It is not a benign condition, which may have a progressive course from the simple steatosis (SS) to nonalcoholic steatohepatitis (NASH), fibrosis or cirrhosis [15]. Along with the clinical course of the NAFLD, obesity, metabolic syndrome and type 2 DM commonly occur [16,17,18]. So far, there is hardly enough data showing how the levels of this “novel liver hormone” are affected in patients with liver disorders [19]. We aimed to measure the betatrophin levels of patients with biopsy proven NAFLD and search for any relationship to the severity of liver injury or the liver enzymes. Also, we compared the betatrophin levels of the NAFLD patients to those of the healthy control subjects.

## 2. Results

**Figure 1:**
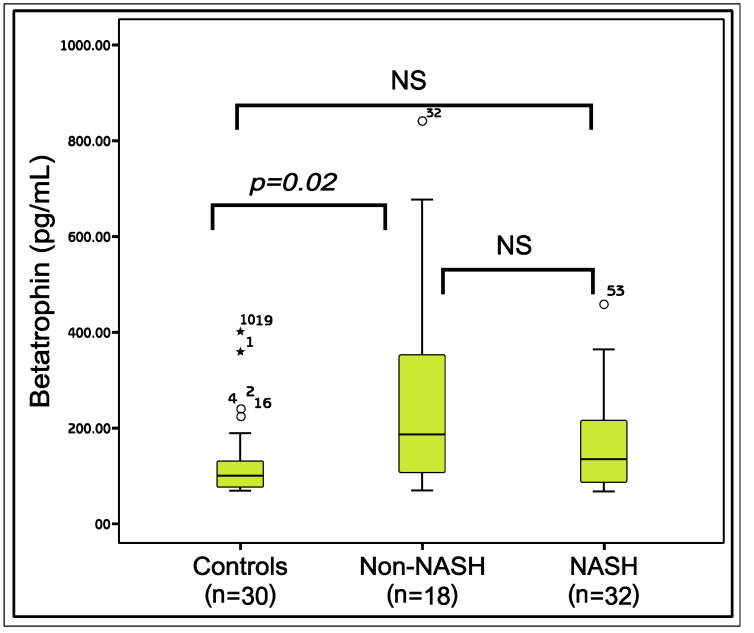
The comparison of the Betatrophin levels between the study groups. Mann-Whitney U test, *p* < 0.05 is significant; NS: Nonsignificant; *: The outliers.

A total of 50 patients with NAFLD [NASH (n = 32; mean age 31.75 ± 5.6), non-NASH (n = 18; mean age 33.38 ± 5.19)] and 30 healthy control subjects (mean age 28.50 ± 3.9) were included. The comparisons of the demographic and the laboratory parameters of the patients and the healthy controls are given in Table 1. According to the results, the mean age (*p* = 0.003), body mass index (BMI), waist circumference (WC), fasting plasma glucose (FPG), insulin, homeostasis model assessment of insulin resistance (HOMA-IR), aspartate aminotransferase (AST), alanine aminotransferase (ALT) (*p* < 0.001 for all) and gamma glutamyl transpeptidase (GGT) (*p* = 0.002), levels of the three groups were significantly different. The difference between the betatrophin levels of the groups was nearly significant (*p* = 0.05). According to the paired comparisons, betatrophin levels in the non-NASH group were significantly higher than the controls (*p* = 0.02). However, the betatrophin levels of the NASH group were not significantly different from the non-NASH (*p* = 0.17) or the control groups (*p* = 0.18) (Figure 1).

Mild fibrosis was present in 36 of the NAFLD subjects. Most of the cases had grade 1 fibrosis (n = 32), while only 3 cases had grade 2 and 1 case had grade 3 fibrosis. No significant difference was present (*p* = 0.46) between the betatrophin levels of the subjects with (167.6 ± 111.0) and without fibrosis (238.3 ± 237.2). According to the univariate correlation analysis, the betatrophin levels were significantly associated with age (r = 0.395, *p* < 0.001), WC (r = 0.230, *p* = 0.04), total cholesterol (TC) (r = 0.349, *p* = 0.002), triglycerides (TG) (r = 0.294, *p* = 0.008), low density lipoprotein cholesterol (LDL-C) (r = 0.388, *p* < 0.001), FPG (r = 0.300, *p* = 0.007), insulin (r = 0.238, *p* = 0.04), HOMA-IR (r = 0.271, *p* = 0.02) and the GGT (r = 0.339, *p* = 0.002) levels. No significant correlation was present between the betatrophin levels and BMI, systolic blood pressure (SBP), diastolic blood pressure (DBP), high density lipoprotein cholesterol (HDL-C), ALT or AST.

The betatrophin levels were also analyzed for any correlation to the main histologic features commonly described in NAFLD. According to the results, the betatrophin levels were significantly and negatively correlated to the steatosis (r = −0.328, *p* = 0.02) and NAFLD activity score (NAS) (r = −0.315, *p* = 0.03). In the stepwise multiple regression analysis, the TG, FPG and NAS were the independent statistically significant predictors of the betatrophin levels [F (3.42) = 11.093, *p* < 0.001] (Table 2 and Table 3).

## 3. Discussion

**Figure 2:**
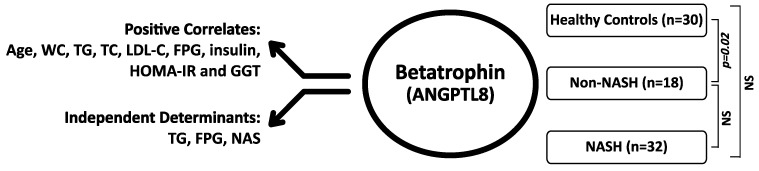
The main findings of the study showing the positive correlates and independent predictors of the Betatrophin levels and the differences between the study groups. Mann-Whitney U test, *p* < 0.05 is significant; NS: Nonsignificant; n: Number; WC: Waist circumference; TG:Triglycerides; TC: Total Cholesterol; LDL-C: Low Density Lipoprotein Cholesterol; FPG: Fasting Blood Glucose; HOMA-IR: Homaistasis model assessment of insulin resistance; GGT: Gamma glutamyl transpeptidase, NAS: NAFLD activity score.

The results show that the NAFLD patients with non-NASH have significantly higher betatrophin levels when compared to those of the healthy control subjects. However, in the more progressed stages, namely in NASH, there is a trend to decrease in the betatrophin levels, with a negative correlation to the severity of liver damage. Among the positive correlates of the plasma betatrophin levels are the demographic parameters (age and WC), lipids (TG, TC and LDL-C), glucose control (FPG, insulin and HOMA-IR) and the liver enzyme GGT. The multivariate analysis indicates that, triglycerides, fasting blood glucose levels, and the severity of liver damage are the independent determinants of the betatrophin levels. The implications of these results are discussed below (Figure 2).

The liver plays an essential role in the metabolism of lipids, carbohydrates and proteins [20]. NAFLD is the most prevalent liver disorder of the Western World [14]. It is not a benign condition as previously assumed, which is usually associated with obesity and metabolic syndrome, and may progress to NASH, fibrosis or less commonly to cirrhosis [15,16]. During the clinical course of the NAFLD, the risk of glucose intolerance and DM significantly increases [17,18,21]. Although several factors, such as inflammation, oxidative stress and pancreatic lipotoxicity may contribute to the impaired glucose metabolism, the exact mechanism of the diabetes risk in NAFLD is not clear [14,17]. A recent study reported that patients with NASH have lower Beta-cell functions, inversely correlated to fibrosis [22]. It is exciting to combine this data with the recent finding of a new liver hormone that controls the lipid and glucose metabolism [6] as it may show the missing piece in the puzzle.

Our results show that betatrophin levels are significantly higher in the early stages of the NAFLD. However, the levels decrease in NASH, in negative correlation to the histological severity of the patients. These results are in accordance with a very recent study, which shows low betatrophin levels in subjects with NAFLD [23]. Betatrophin levels were the independent predictors of NASH and fibrosis in this study. Regarding the significant correlation of betatrophin with the insulin levels in our study, these findings are important as they may explain the increased frequency of diabetes along with the course of NAFLD. Serum triglycerides and fasting glucose levels appear to be the significant positive predictors of the betatrophin levels in this study. Regarding the role of betatrophin in modulating beta cell functions [6], fatty acid metabolism and lipid homeostasis [2,3,4], it would be tempting to consider that elevation of betatrophin levels in the early stages of NAFLD may be a self defense of liver in response to cardiovascular and metabolic offenses such as aging, obesity, dyslipidemia, insulin resistance or glucose intolerance [24,25].

The previous data about the circulating levels of betatrophin in metabolic disorders are equivocal. Some studies in patients with type 2 DM have yielded elevated [8,9,10,11], while others reported unaltered [12] or decreased betatrophin levels [13]. Likewise, the reports about the betatrophin levels in the obese subjects are controversial [9,12,13]. The data regarding the relationship of the betatrophin levels to the serum lipids, insulin sensitivity, fasting glucose and the measures of adiposity are also not clear [7,8,9,10,11,12,13]. None of these studies mentioned whether any of the study participants had NAFLD or not. However, regarding the high prevalence of NAFLD in patients with dyslipidemia, obesity and type 2 DM [16,17,18], it is reasonable to assume that many of the patients in the previous reports had NAFLD, which could have affected the previous results. Another reason of the discrepancies in these studies might be the use of different betatrophin ELISA kits in different studies [23]. Depending on the selected antibodies against either the N- or the C-terminus of the protein, the proteolytic regulation of betatrophin may result in different circulating levels by different ELISA kits [26]. Mass Spectrometry with labeled standard peptides is a much better protocol to measure betatrophin content in serum or liver.

There may be several limitations of this study. The linear regression analysis shows that the severity of liver damage is an independent predictor of the betatrophin levels. However, the cross-sectional design of the study precludes further comments on the causality between the liver damage and betatrophin levels. We would expect that the betatrophin levels differ between the NAFLD subjects with NASH and non-NASH. The small size of the study population and the high standard deviations could be the reason for the lack of significant difference between these groups. Also, histologically most of the NASH patients were not at the advanced stages of the liver damage, which may be another reason for the lack of difference between the betatrophin levels of the groups. Despite these limitations, the study has significant advantages. All the NAFLD cases were confirmed by biopsies, which helps us better identify the effect of histological severity on the betatrophin levels. Also, the confounding factors for betatrophin were eliminated in this study as none of the patients had type 2 DM or were under any medication for any metabolic disorder.

## 4. Materials and Methods

### 4.1. Study Design and Population

The study was conducted in Gulhane Military Medical Academy, the tertiary medical center of the Turkish Army. Liver enzyme elevations are often observed in the periodic Health controls of the officers and warrant officers. Fine Needle Liver Biopsy may be performed to those with recurrent or persistent enzyme elevations. Therefore, the study population described below, consists only of young male patients.

The study was performed by using the plasma samples of 50 male subjects with biopsy-proven NAFLD and 30 healthy male controls. Inclusion criteria were persistently (at least for 6 months) elevated aminotransferases; ultrasonographic presence of bright liver without any other liver or biliary tract disease; liver histology compatible with a diagnosis of NAFLD. Patients were excluded if they had a history of alcohol consumption >140 g/wk, as assessed by a detailed interview extended to family members; positive testing for hepatitis B virus or hepatitis C virus, BMI ≥ 40 kg/m^2^; positive serum markers of autoimmune or celiac disease; abnormal copper metabolism or thyroid function tests; a diagnosis of overt DM [FPG ≥ 126 mg/dL or ≥200 mg/dL at 2 h on a standard oral glucose tolerance test, (OGTT)] and systemic hypertension; exposure to occupational hepatotoxins or drugs known to be steatogenic or to affect glucose and lipid metabolism. The study was approved by the local ethics committee of Gulhane School of Medicine (50687469-1491-318-14/1648.4-824) and registered to the ClinicalTrials.gov (NCT02176811). All participants gave written informed consents to study, which was conducted according to the Helsinki Declaration.

### 4.2. Clinical and Laboratory Data

All participants provided a medical history and underwent a clinical examination. The weight and height of the participants were measured with a calibrated scale after the patients had removed their shoes and any heavy clothing. BMI was computed as body weight/height^2^. WC was measured as the mid-point between the lower costal margin and the level of the anterior superior iliac crests.

Fasting blood specimens were collected from all participants after an 8-h overnight fast. The samples were centrifuged for 15 min at 4000 rpm, aliquoted and immediately frozen at −80 °C for analyses until examination. All samples were run in the same assay. FPG, TC, TG, and HDL-C levels were measured by the enzymatic colorimetric method with Olympus AU2700 auto analyzer using reagents from Olympus Diagnostics, (GmbH, Hamburg, Germany). LDL-C was calculated by Fridewald’s formula [27]. The serum basal insulin level was measured in duplicate by the chemiluminescence’s method using reagents from Roche Diagnostics (Mannheim, Germany). Insulin resistance was calculated by modified HOMA-IR index, with the following formula: HOMA-IR = fasting plasma insulin (μU/mL) × FPG (mg/dL)/ 405. A 2-h OGTT was performed in each patient with the standard 75 g of glucose. Glucose tolerance status was determined according to the classification of the American Diabetes Association (ADA) in which FPG levels up to 99 mg/dL are considered normal and DM is defined by a FPG level of 126 mg/dL or greater, or a 2-h plasma glucose levels of 200 mg/dL or greater [28].

Plasma betatrophin levels were determined by enzyme-linked immunosorbent assay (ELISA) (Human Human Hepatocellular carcinoma-associated protein TD26 ELISA Kit, EIAab Science Co. Ltd. Catalog no: E11644H; Wuhan, China) according to the manufacturer’s protocol. Measurements were carried out using ELISA plate reader Bio-Tek Synergy HT (Biotek Instruments Inc., Winooski, VT, USA).

### 4.3. Liver Histology

Liver biopsy specimens, routinely processed and analyzed as described elsewhere, were at least 20 mm long and read by the same liver pathologist who was unaware of the patient’s clinical and laboratory data. The main histologic features commonly described in NAFLD, including steatosis, inflammation (portal and lobular), hepatocyte ballooning and fibrosis, were scored by using the semi quantitative classification of Kleiner et al. [29]. Features of steatosis, lobular inflammation, and hepatocyte ballooning were combined in a score ranging from 0 to 8, the NAFLD activity score (NAS). Cases with NAS ≥ 5 are diagnostic of NASH, cases with NAS ≤ 2 are diagnostic of SS, and cases with scores in between are considered as borderline NASH. For statistical purposes, the borderline NASH and SS cases were combined as the Non NASH category in this study.

### 4.4. Statistical Analysis

All data were recorded on a computer database and analyzed using SPS 15.0 package program (SPSS, Inc., Chicago, IL, USA). Results are expressed as mean ± S.D. when normally distributed and as median (range) when non-normally distributed. The variables were assessed for normality using Kolmogorov-Smirnov test. Then the Levene’s test was used to evaluate the equality of variance. The multiple comparisons between the groups were performed by ANOVA and Kruskal Wallis tests. Paired comparisons of independent groups were performed by using Student’s t-test and Mann–Whitney U test as appropriate. The correlation analyzes were done combining all of the individuals and using Pearson or Spearman methods. We used stepwise multiple regression test in order to find out the independent determinants of plasma betatrophin levels. The NAS in addition to the independent variables, which have significant correlation with betatrophin were included in the multiple regression model. The *p* value of <0.05 was taken as a threshold of statistical significance.

## 5. Conclusions and Recommendations

In summary, the results of the present study show that the betatrophin levels significantly increase in the early stages of NAFLD. However, the levels decrease in the more progressed stage, in NASH. Also the results show that betatrophin levels are in close association with aging, visceral obesity, insulin resistance, glucose, trglycerides and GGT levels. The above findings together with the negative predictive role of liver histology on betatrophin levels may produce an important mechanistic finding to understand the cause of increased frequency of type 2 DM during the course of NAFLD. Still, these findings produce more questions than the answers. How circulating betatrophin levels are affected in the advanced stages of NAFLD? Is there a role of liver betatrophin secretion kinetics in the pathogenesis of glucose intolerance and diabetes mellitus? Is there any association between the betatrophin levels and the risk of cardiovascular morbidity and mortality? Further, prospective studies are warranted to give answers to these questions and to better understand the role of this novel liver hormone in the pathogenesis of chronic metabolic disorders.

## Figures and Tables

**Table 1:** The Comparison of the demographic and the laboratory parameters of the patients with NAFLD and the healthy controls.

	Healthy Controls (n = 30)	Patients with NAFLD		*p* Value Between Three Groups	*p* Value Control vs. Non-NASH	*p* Value Control vs. NASH	*p* Value Non-NASH vs. NASH
		non-NASH (n = 18)	NASH (n = 32)				
**Age (years)**	28.50 ± 3.91	33.38 ± 5.19	31.75 ± 5.6	**0.003**	**0.001**	**0.01**	0.31
**BMI (kg/m^2^)**	24.91 ± 1.44	29.26 ± 2.26	27.85 ± 2.03	**<0.001**	**<0.001**	**<0.001**	**0.03**
**WC (cm)**	88.50 ± 5.33	101.13 ± 6.14	100.43 ± 5.68	**<0.001**	**<0.001**	**<0.001**	**0.52**
**SBP (mmHg)**	114.66 ± 7.30	113.33 ± 7.66	114.68 ± 10.15	0.845	0.55	0.99	0.62
**DBP (mmHg)**	71.66 ± 7.91	70.55 ± 8.02	71.56 ± 6.77	0.870	0.64	0.96	0.64
**FPG (mg/dL)**	78.90 ± 8.46	94.61 ± 12.92	94.18 ± 9.36	**<0.001**	**<0.001**	**<0.001**	0.89
**TC (mg/dL)**	184.75 ± 29.28	188.55 ± 39.95	194.53 ± 48.0	0.634	0.71	0.35	0.66
**TG (mg/dL)**	124 (55–290)	146 (49–525)	143 (26–472)	0.182*	0.10	0.03	0.74
**HDL-C (mg/dL)**	43.86 ± 6.16	43.05 ± 7.5	40.71 ± 7.36	0.204	0.70	0.08	0.30
**LDL-C (mg/dL)**	114.67 ± 28.38	117.76 ± 33.54	115.56 ± 37.16	0.955	0.74	0.92	0.84
**AST (IU/L)**	23.00 ± 4.46	43.44 ± 10.79	57.75 ± 21.42	**<0.001**	**<0.001**	**<0.001**	**0.01**
**ALT (IU/L)**	22.03 ± 7.03	90.33 ± 30.79	123.72 ± 47.80	**<0.001**	**<0.001**	**<0.001**	**0.01**
**GGT (IU/L)**	22.0 (20–30)	50.5 (24–118)	61.0 (20–455)	0.002 *	**<0.001**	**0.001**	0.53
**Insulin (µU/mL)**	6.44 (2.2–10.9)	15.25 (4.90–32.34)	12.48 (2.57–45.13)	<0.001 *	<0.001 **	<0.001 **	0.76 **
**HOMA-IR**	1.24 (0.41–2.5)	3.68 (0.86–7.19)	2.96 (0.53–11.03)	<0.001 *	<0.001 **	<0.001 **	0.69 **
**Betatrophin (pg/mL)**	87.17 (60.1–131.2)	175.12 (56.1–839.1)	122.28 (53.1–450.1)	0.05 *	0.02 **	0.18 **	0.17 **

The data is given in mean ± SD or median (25%–75%). Between group comparisons were made by One-way ANOVA. Post-hoc comparisons were made by Independent Samples *t*-test, Kruskal-Wallis * and Mann-Whitney U ** tests. BMI: body mass index, WC: waist circumference, SBP: sistolic blood pressure, DBP: diastolic blood pressure, FPG: fasting plasma glucose, TC: total cholesterol, TG: triglyceride, HDL-C: high-density lipoprotein cholesterol, LDL-C: low-density lipoprotein cholesterol, AST: aspartate Aminotransferase ALT: alanine Aminotransferase, GGT: gamma-glutamyl transpeptidase HOMA-IR: homeostatic model assessment-insulin resistance, NAFLD: nonalcoholic fatty liver disease, NASH: nonalcoholic steatohepatitis.

**Table 2:** Stepwise multiple regression test results depicting the independent predictors of serum betatrophin level.

	MODEL 1* (R = 0.534)		MODEL 2* (R = 0.613)		MODEL 3* (R = 0.665)	
	Beta **	*p*	Beta **	*p*	Beta **	*p*
**Glucose**	0.308	0.017				
**NAS**	−0.288	0.022	−0.260	0.030		
**WC**	0.111	0.393	0.148	228	0.107	0.370
**Age**	0.271	0.042	0.178	0.196	0.136	0.312
**TC**	0.093	0.615	0.071	0.685	0.071	0.670
**LDL-C**	0.234	0.139	0.225	0.134	0.188	0.193
**GGT**	0.126	0.336	0.067	0.599	0.089	0.467
**Insulin**	0.024	0.856	−0.078	0.545	−0.086	0.488
**HOMA-IR**	0.087	0.505	−0.061	0.658	−0.070	0.598

NAS: nonalcoholic fatty liver disease activity score, WC: waist circumference, TC: total cholesterol, LDL-C: low density lipoprotein cholesterol, GGT: gama glutamyl transpeptidase, HOMA-IR: homeostasis model assessment of insulin resistance. In Model 1 only the triglyceride, in Model 2 the trigliyceride and glucose and in Model 3 the triglyveride, glocose and NAS were the constant variables. ** Beta represents the “standardized coefficients”.

**Table 3:** Independent predictors of betatrophin according to the Stepwise Multiple Regression Test.

	Unstandardized Coefficients		Standardized Coefficients	*p*
	B	Standard Error	Beta	
**Consant**	−235.097	220.104		
**TG**	0.668	0.173	0.457	0.000
**Glucose**	5.088	2.159	0.281	0.023
**NAS**	−36.560	16.305	−0.260	0.030

TG: triglyceride, NAS: nonalcoholic fatty liver disease activity score.

## Data Availability

All data are available in manuscript.
